# A Magnetic Field Sensor Based on Directional Coupling in a Magnetic Fluid-Filled Photonic Crystal Fiber

**DOI:** 10.3390/ma16175805

**Published:** 2023-08-24

**Authors:** Yingchao Liu, Lijun Zhang, Shuang Ren, Hailiang Chen

**Affiliations:** 1 Key Laboratory of Industrial Intelligent Perception, School of Artificial Intelligence, North China University of Science and Technology, Tangshan 063210, China; yingchaoliucbb@163.com (Y.L.); 15630433935@163.com (L.Z.); renshuang0326@163.com (S.R.); 2 State Key Laboratory of Metastable Materials Science & Technology, School of Science, Yanshan University, Qinhuangdao 066004, China; 3 Key Laboratory for Microstructural Material Physics of Hebei Province, School of Science, Yanshan University, Qinhuangdao 066004, China

**Keywords:** magnetic field sensor, photonic crystal fiber, magnetic fluid, modes coupling

## Abstract

In this paper, a dual-core photonic crystal fiber (DC-PCF) sensitivity sensor filled with magnetic liquid is introduced and investigated with the finite element method (FEM). To regulate the energy coupling involving the two cores, the magnetic fluid is filled into the pore between the two cores. To adjust the coupling between the supermodes in the DC-PCF, the refractive index (RI) of the air hole filled magnetic fluid may change due to the external magnetic field. This specifically created a magnetic fluid-filled DC-PCF; the magnetic fluid-filled hole is not used as the core for energy transmission, thus avoiding transmission loss. The dip wavelength and the magnetic field displayed an excellent linear connection between 80 and 260 Oe, depending on the numerical data. The detection sensitivity of the magnetic field reached 515.75 pm/Oe at a short fiber length of 482 µm. The designed magnetic fluid-filled DC-PCF has high sensitivity and small volume and has great application prospects in magnetic field detection in the medical and industrial fields.

## 1. Introduction

With the advancement of modern technologies, magnetic field sensors have become increasingly significant. They are widely used in biomedical detection, the aerospace industry and safety monitoring [1,2,3]. Traditional methods often use Hall effects, magnetic transistors, and magnetoresistance to sense magnetic fields [4,5]; however, these methods have low sensitivity, complex structure, and high cost. A magnetic field sensor based on a photonic crystal fiber (PCF) provides benefits over conventional techniques, including better sensitivity, linear response, anti-interference capability, small size, and low power consumption, which is suitable for the application of high-precision, reliable and stable measurement of magnetic fields.

A novel kind of functional material is called magnetic fluid (MF). MFs are not used as cores for energy transfer, and when a hole filled with a magnetic fluid is used as an energy transfer core, the magnetic fluid generates friction, eddy currents, and hysteresis losses, among other things, during the transfer process. These losses result in energy loss and reduce the efficiency of energy transfers. Therefore, the MF core modulates the coupling effect to alter the sensitivity in response to changes in the external magnetic field. MF is a stable colloidal liquid, it not only has the fluidity of liquid, which is conducive to integration into the pores of microstructured fibers, but also has the magnetism of magnetic particles [6]. The optical fiber designed in this paper has a solid-core structure, and the coupling efficiency between solid-core materials is usually limited by the difference in their magnetic permeability and the distance. By introducing an MF core between two solid cores, its tunable magnetism can optimize the magnetic coupling between the cores and thus improve the coupling efficiency. The magnetic nanoparticles can be prepared by chemical coprecipitation [7]. The magnetic nanoparticles surrounded by surfactants are evenly dispersed in the liquid carriers. The relationship between the magnetic particle concentration and the RI of MF is linear. When the external magnetic field intensity (MFI) surpasses a certain threshold, the correlation between the magnetic field (MF) and the refractive index (RI) of the magnetic fluid adheres to the Langevin equation [8]. Owing to the magneto-optical characteristics of MF, there have been various suggested MF-based photonic devices, including optical switches, filters, and magnetic field sensors [9,10,11,12].

Knight, J.C. et al. created the first PCF, also known as microstructured fiber or holey fiber, in 1996 [13].

The emergence of PCF has brought a major breakthrough for fiber technology. Wang and colleagues used ethanol and magnetic fluid in the fiber’s air to construct a PCF-based temperature and magnetic field sensor with a high sensitivity of 18.37 nm/Oe [14]. An innovative PCF magnetic field sensor was created by Huang et al. According to the experimental findings, the magnetic field sensitivity may be as high as 61.25 pm/Oe between 50 and 130 Oe [15]. According to experiments, Wang et al. developed a dual parameter sensor for temperature and MF measurements. The sensor is based on filling a photonic crystal fiber (PCF) with magnetic flux material, with polyethylene glycol serving as a surfactant. The magnetic field sensor demonstrates an impressive sensitivity of up to 924.63 pm/mT [16]. Xu et al. created a unique PCF magnetic field sensor that included MF in its two elliptic center holes. Numerical simulation results show that the maximum sensitivity of the sensor can be close to 1200 pm/Oe [17]. However, the previously reported magnetic geld sensors have low sensitivity, and the structure of PCF was usually complex and less flexible. We propose a kind of DC-PCF magnetic field sensor. Compared to traditional PCF, DC-PCF has significant advantages in security, transmission capacity, transmission loss, flexibility and versatility, which will significantly advance the development of optical technology and fiber communication. Even in some PCFs, light is transmitted in the high loss magnetic fluid. It is essential to investigate a DC-PCF high sensitivity magnetic field sensor and vitally light transmission in silica core.

In this paper, a magnetic field sensor, an MF-filled DC-PCF using the FEM, was examined. The problem domain is divided into finite elements by the FEM, which are connected by nodes. The governing equations are approximated and numerically solved on these finite elements. The accuracy depends on the mesh density, material properties and the choice of boundary conditions. Validation is often required to establish confidence in numerical results. Only one central air hole in the DC-PCF was designed to be infiltrated with MF. The mode coupling effect between the supermodes could be adjusted by the external magnetic field. Calculations revealed that the measurement sensitivity was 515.75 pm/Oe. Wavelength shift and magnetic MFI have a linear relationship between them between 80 and 260 Oe, which made it a significant candidate in the field of magnetic field detection.

## 2. MF-Filled DC-PCF and Operation Principles

**Figure 1:**
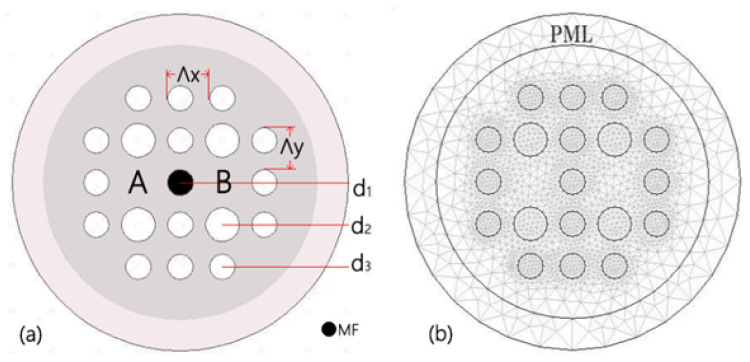
(**a**) Schematic diagram of the designed cross-section of the DC-PCF filled with MF. (**b**) FEM mesh and PML.

Figure 1a depicts the cross-section of the MF-filled DC-PCF. The cladding’s pores are placed in a lattice pattern that is rectangular. Compared with hexagonal distribution, a rectangular arrangement of holes can generate greater mode birefringence [18]. Depicting larger mode birefringence leads to shorter coupling lengths. The DC-PCF magnetic field sensor may be made significantly smaller as a result. The horizontal hole spacing of surrounding voids are indicated by $\Lambda_{x}$, and $\Lambda_{y}$ represents the spacing in the vertical direction, respectively. Both the values of $\Lambda_{x}$ and $\Lambda_{y}$ are set at 2 µm. The diameters of the tiny white, huge white, and tiny black air holes are shown by d${}_{1}$, d${}_{2}$ and d${}_{3}$, respectively. These diameters are initially set at d${}_{1}$ = 1.2 µm, d${}_{2}$ = 1.6 µm and d${}_{3}$ = 1.2 µm, respectively. It is well known that the efficient refractive indices of core modes are mainly influenced by its most adjacent air holes [19]. To achieve magnetic field detection, the center air hole is fashioned as an MF infiltration hole. The filling technology demonstrated by [20,21] can be used to achieve the selective infilling of the MF. Figure 1b depicts the meshed cross-section of the DC-PCF. The energy of the scattered light is absorbed using a Perfectly Matched Layer (PML). The scattering boundary condition (SBC) acts as the PML’s outer border to further boost the absorption of scattering light. COMSOL Multiphysics 3.5a software meshes the computational region using free triangles. The size of the grid has an effect on the accuracy and efficiency of the solution results. Smaller grids can more accurately capture the details and variations in the problem domain, but add complexity and cost to the calculation. Larger grids can reduce computation time and cost, but may result in less precision of the results. Under the premise that the calculation accuracy meets the requirements, we use coarse mesh division to improve the calculation efficiency. The DC-PCF is meshed into about 16,814 elements. In the designed DC-PCF, the RI of air is set as *n*${}_{air}$ = 1. The DC-PCF’s background material is fused silica glass, and the Sellmeier equation may be used to calculate its material dispersion [22].

(1) $$n^{2}\left(\lambda \right)=1+\frac{m_{1}\lambda^{2}}{\lambda^{2}-k_{1}{}^{2}}+\frac{m_{2}\lambda^{2}}{\lambda^{2}-k_{2}{}^{2}}+\frac{m_{3}\lambda^{2}}{\lambda^{2}-k_{3}{}^{2}}$$

λ stands for wavelength in micrometers. The parameters in Equation (1) are *m* ${}_{1}$ = 0.6961663, *m*${}_{2}$ = 0.4079426, *m*${}_{3}$ = 0.8974794, *k*${}_{1}$ = 0.0684043 µm, *k*${}_{2}$ = 0.1162414 µm, and *k*${}_{3}$ = 9.896161 µm. The Langevin function is followed by the refractive index of the MF [23]:

(2) $$n\left(H,T\right)=\left[n_{t}-n_{i}\right]\left[\coth\left(\alpha \frac{H-H_{i,n}}{T}\right)-\frac{T}{\alpha \left(H-H_{i,n}\right)}\right]+n_{t},\left(H>H_{i,n}\right)$$

where *n*(*H*, *T*) is the RI of the MF, which, within a particular range of magnetic field intensity, fluctuates with temperature and MF, *n*${}_{t}$ is RI value as MF reaches the saturation magnetization, and the magnetic field’s threshold value is *H*${}_{i,n}$. The RI of MF is *n*${}_{i}$ because the magnetic field *H* is smaller than *H*${}_{i,n}$. The temperature in Kelvin is *T*. α denotes the coefficient of the fitting.

**Figure 2:**
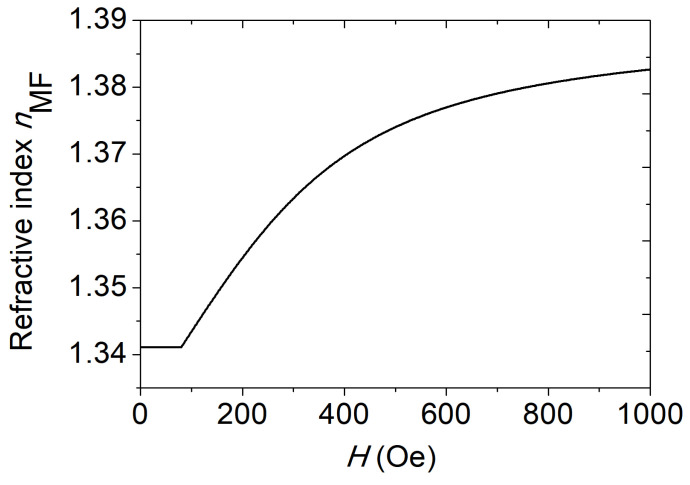
The RI of MF variation with magnetic field.

In this paper, the MF is carried by water as its liquid carrier, and its concentration is 25%. The parameters are *H*${}_{i,n}$ = 80 Oe, *n*${}_{i}$ = 1.3411, *n*${}_{t}$ = 1.3901, α = 0.143, and *T* = 293 K [23]. In Figure 2, the MF strength H and the RI of the MF variation are displayed.

**Figure 3:**
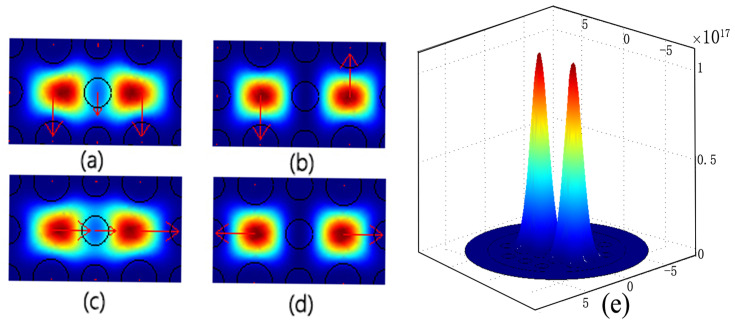
Electric filed distributions of the four supermodes, the red arrow represents the direction of the electric field. (**a**) Even mode with a polarization of y, (**b**) odd mode with a polarization of y, (**c**) even mode with a polarization of x, and (**d**) odd mode with a polarization of x, (**e**) 3-D electric field distributions of the supermode at the coupled wavelength 1.55 µm.

The two silica cores beside the central MF-filled hole in DC-PCF form two independent waveguides. According to the supermodes theory, there are four supermodes, which, in the x and y polarization directions, respectively, comprise odd mode and even mode. The model properties of the four supermodes are calculated using COMSOL Multiphysics 3.5a software. Figure 3 displays the 2-D and 3-D electrical field distributions at 1.55 µm wavelength.

The coupling length (*L*${}_{i}$) between the supermodes can be described as [24]:

(3) $$L_{i}=\frac{\pi }{\left|\beta_{e}^{i}-\beta_{o}^{i}\right|}=\frac{\pi }{2\left|n_{e}^{i}-n_{o}^{i}\right|},i=x,y$$

where $\beta_{e}^{i}$, $\beta_{o}^{i}$ are the propagation constants and $n_{e}^{i}$, $n_{o}^{i}$ are the actual refractive indices. The odd and even supermodes are represented by *o* and *e*, respectively. *I* represents the polarized direction. The parameters are set as *d*${}_{1}$ = 1.2 µm, *d*${}_{2}$ = 1.6 µm, *d*${}_{3}$ = 1.2 µm, $\Lambda_{x}$ = $\Lambda_{y}$ = 2 µm and *H* = 80 Oe. The thickness of PML is 2 µm, and its RI is 0.03 higher than that of silicon. The four fundamental simulated real refractive indices at a wavelength of 1.55 µm are $n_{e}^{x}$ =1.4110976, $n_{o}^{x}$ = 1.406277541, $n_{e}^{y}$ = 1.409642134 and $n_{o}^{y}$ = 1.404987619. The coupling distances calculated by Equation (3) are 160.8 µm and 166.5 µm for the two polarized directions that are orthogonal. Coupling length is a vital parameter for DC-PCF. Power may be transferred from one fiber core to another thanks to the connection length. It is thought that the light is fired at the input ports of cores A. The normalized power in the input ports of cores A and B is set at 1 and 0, respectively [25]. You can define the normalized power at the output port of A as [26]:

(4) $$NP_{i}=\frac{P_{i-\;\mathrm{out}\;}}{P_{\mathrm{in}\;}}=\cos^{2}\left(\frac{\pi L}{2L_{i}}\right),i=x,y$$

where *NP*${}_{i}$ is the normalized output power. *P*${}_{\mathrm{in}\;}$ = 1 and *P*${}_{i-\;\mathrm{out}\;}$ are the power of incident light and output light at core A, respectively. *L* represents the length of MF-filled DC-PCF. It is possible to compute the output power in dB:

(5) $$T_{i}=10\log_{10}\left(NP_{i}\right),\;i=x,y$$

The transmission spectrum fluctuates as a result of variations in supermode refractive indices and magnetic field changes. Using the directional coupling principle, the wavelength of the dip may be monitored to determine the magnetic field. The following equation can be used to determine the sensitivity *S*(*H*).

(6) $$S\left(H\right)=\frac{\Delta \lambda_{\mathrm{dip}\;}}{\Delta H}$$

ΔH is the fluctuation in MFI, while $\Delta \lambda_{\mathrm{dip}\;}$ is the dip wavelength shift.

## 3. Results and Discussion

**Figure 4:**
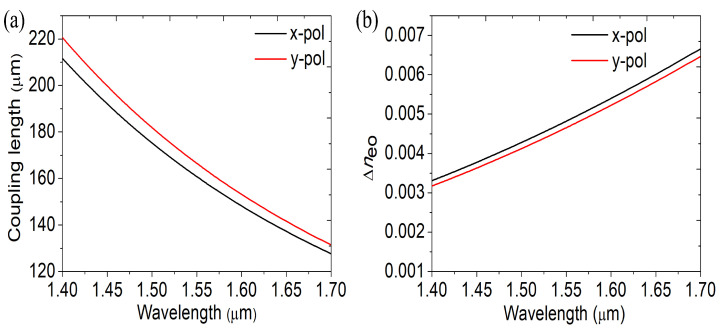
(**a**) Coupling length and as a function of wavelength, (**b**) the difference in the RI of supermodes. The magnetic field is set at 80 Oe.

According to Equation (3), it is determined that there is a wavelength link between supermode coupling length and refractive index. Figure 4a,b depicts, respectively, how coupling length and RI difference change with wavelength. At 80 Oe, the magnetic field’s strength is determined. The discrepancy in $\Delta n_{eo}$’s RI grows as wavelength increases. The coupling lengths thus shorten in both orthogonal directions. Moreover, y-polarization direction coupling length is more significant than in the x-polarization direction.

**Figure 5:**
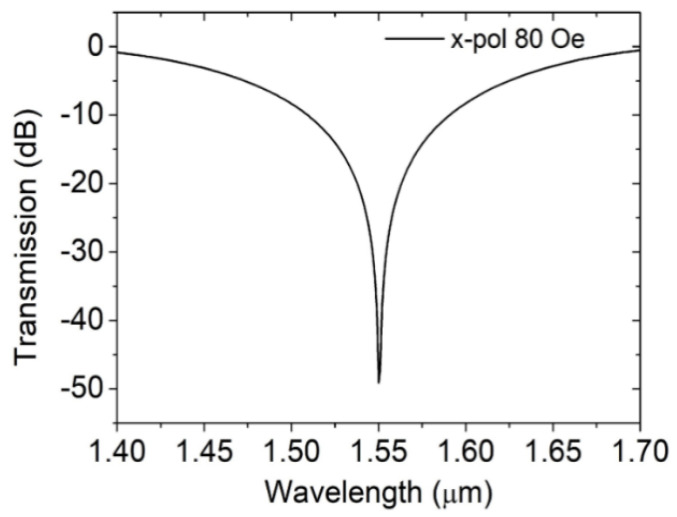
Transmission spectrum in x-polarized direction. The magnetic field and the DC-PCF length are 80 Oe and 482 µm, respectively.

According to Equations (4) and (5), the transmission spectrum is correlated with both the DC-PCF length and coupling length. Since 1.55 µm is usually the communication wavelength, many devices are designed at 1.55 µm. Moreover, we use 1.55 µm as the reference wavelength. Figure 5 displays the transmission spectrum of x polarization mode. The DC-PCF length and magnetic field are set as 482 µm and 80 Oe. At the 1.55 µm communication window, a visible depression forms. Then, theoretical analysis is performed on the DC-PCF’s MF’s sensing properties.

**Figure 6:**
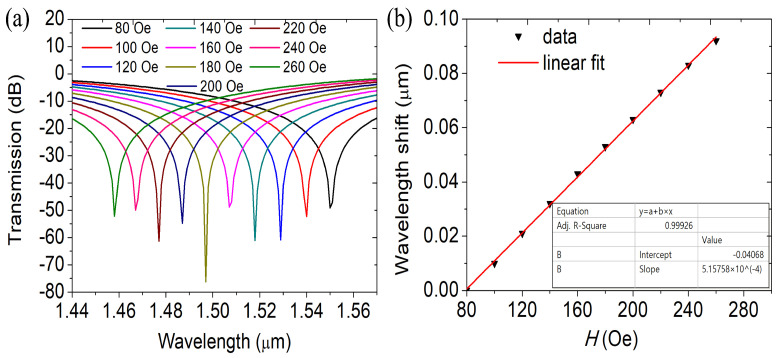
(**a**) Transmission spectra at different MFI and (**b**) the fluctuation of the dip wavelength shift with magnetic field.

As the magnetic field is stronger, the MF’s RI rises. The RI of the supermodes differ correspondingly. Figure 6a displays transmission spectra at varied MFI, whereas Figure 6b displays variations in dip wavelength shift with the MF. When the MFI increases from 80 to 260, the dip wavelength advances toward the blue end of the spectrum. Because of this, the coupling wavelength is sensitive to variations in the MFI, such that the MFI may be approximately determined by in the transmission spectrum, measuring the coupling wavelength.

The MFI and the coupling wavelength are inversely correlated, as shown in Figure 6b. The relation curve of the two is fitted, and the slope and fitting variance can be obtained. The physical meaning of the slope is the DC-PCF magnetic field sensor’s sensitivity, and the sensor’s sensitivity is 515.75 pm/Oe. The significance of the fitted variance is to assess the quality or accuracy of the fitted linear relationship. The fit variance ranges from 0 to 1, with better the consistency of the fit results with the actual data closer to 1. The MFI and the coupling wavelength exhibit a strong linear relationship, as shown by the fitting variance of 0.99926. It offers many benefits and lowers the measuring error of magnetic fields as an MF sensor. We generated and examined the transmission spectra at various structural parameters to investigate the impact of the PCF’s structural factors on magnetic field detection performance.

**Figure 7:**
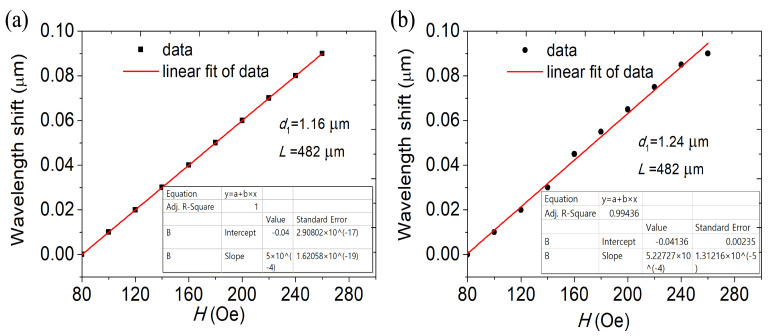
The variation in the dip wavelength shift with MFI at (**a**) *d*${}_{1}$= 1.16 µm and (**b**) *d*${}_{1}$= 1.24 µm. *L* = 482 µm.

**Figure 8:**
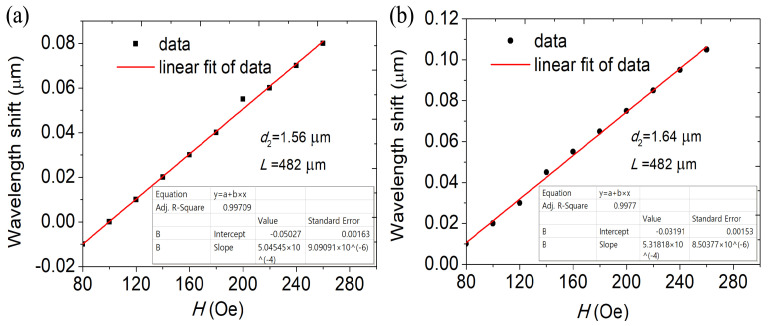
The variation in the shift of dip wavelength with MFI at (**a**) *d*${}_{2}$ = 1.56 µm and (**b**) *d*${}_{2}$ = 1.64 µm. *L* = 482 µm.

Figure 7 demonstrates the change as a function of the dip diameter’s change in wavelength *d*${}_{1}$ of the MF. The DC-PCF length has been adjusted at *L* = 482 µm. When *d*${}_{1}$ increases, the volume of the filled magnetic fluid increases, resulting in the average decrease of the effective RI in the propagation path of light, the effective RI the four modes becomes smaller, the discrepancy between the effective RI of the four supermodes and those indices also decreases, which means that the mutual coupling between the different supermodes becomes more uniform and consistent. The coupling length lengthens as *d*${}_{1}$ rises, and the increase in coupling length can provide more coupling opportunities, so that more energy is coupled from one transmission mode to another fiber mode, which will enhance the strength of the dual-core coupling and improve the energy transmission efficiency. The slopes of the curves control how sensitive the MFI sensor is. The simulation outcomes show that the MFI sensor’s sensitivity rises as diameter *d*${}_{1}$ grows. The sensitivity is 500, 515.75 and 522.72 pm/Oe as *d*${}_{1}$ is 1.16, 1.20 and 1.24 µm, respectively, as can be seen by comparing Figure 6b and Figure 8. As *d*${}_{1}$ rises, the sensitivity rises as well. The interaction between the transmitted light in the fiber cores and the magnetic fluid is improved with an increase in coupling length, enhancing sensitivity.

Dip wavelength offset varying with the MFI at different diameters *d*${}_{2}$ is shown in Figure 8. DC-PCF length is set as *L* = 482 µm. The difference between the effective RI of the supermodes increases as the effective RI of the four supermodes decrease and as *d*${}_{2}$ increases. As a result, the coupling length shortens with increasing *d*${}_{2}$, and a blue shift in the wave trough is seen. The sensitivity of the DC-PCF sensor greatly rises as the diameter *d*${}_{2}$ rises from 1.56 to 1.64 µm, as can be shown in Figure 6b and Figure 8. The sensitivity is 504.54, 515.75 and 531.81 pm/Oe for *d*${}_{2}$ = 1.56, 1.60 and 1.64 µm, respectively. The transmitted light in the core is first compressed as *d*${}_{2}$ rises, enhancing the sensitivity and mode coupling strength.

**Figure 9:**
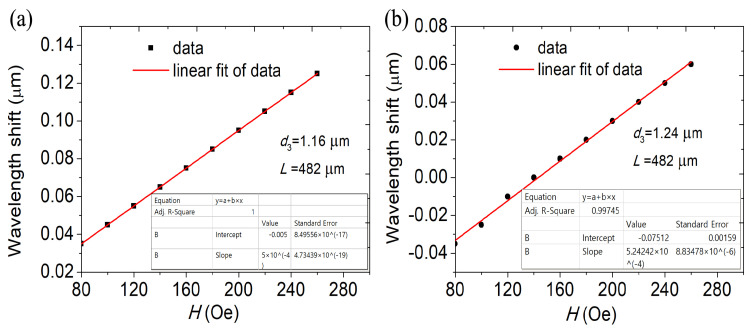
The variation in the shift of dip wavelength with MFI at (**a**) *d*${}_{3}$ = 1.16 µm and (**b**) *d*${}_{3}$ = 1.24 µm. *L* = 482 µm.

The dip wavelength offset is shown in Figure 9 as a function of the MFI at various *d*${}_{3}$ diameters. The length of the DC-PCF is fixed to *L* = 482 µm. Since *d*${}_{3}$ is the hole that magnetic fluid fills, as *d*${}_{3}$ increases, so does the magnetic fluid region. When *d*${}_{3}$ increases, there is a decreasing disparity between the effective RI of the four supermodes and their combined effective refractive indices. The coupling length lengthens as a result, the wave trough undergoes a red shift, and the sensitivity is improved. As *d*${}_{3}$ = 1.24 µm, the sensitivity increases to 524.24 pm/Oe.

**Figure 10:**
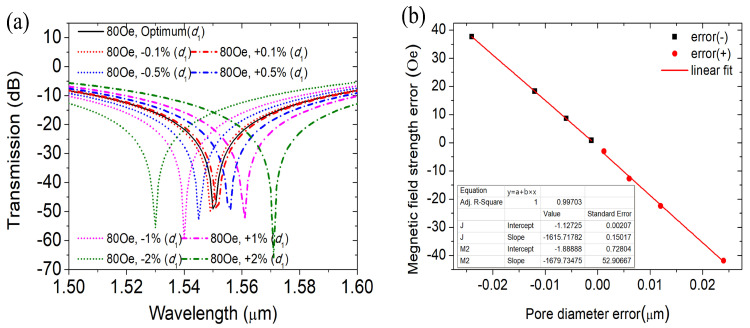
(**a**) Transmission spectra at different errors of *d*${}_{1}$ and (**b**) the relationship between the error of *d*${}_{1}$ and magnetic field error.

When the fabrication error of the air hole filled with MF is −0.1% *d*${}_{1}$, the coupling wavelength under the magnetic field 80 Oe moves from 1.55 µm to 1.549 µm, and the measured MFI is 80.81 Oe with an error of 0.81, which is within the acceptable range. When the error increases from −0.1% *d*${}_{1}$ to −2% *d*${}_{1}$, the coupling wavelength moves to the short wavelength in turn. When the preparation error is −2% *d*${}_{1}$, the magnetic field intensity error reaches 37.65 Oe, which is beyond the acceptable range. There is a good linear relationship between the magnetic field measurement error and the air hole diameter error y = −1615.7178x − 1.12725, as shown in Figure 10b, $R^{2}$ = 1. Similarly, when the preparation error increases from +0.1% *d*${}_{1}$ to +2% *d*${}_{1}$, the coupling wavelength is redshifted from 1.551 µm to 1.571 µm. The measurement error of the corresponding MFI increased from 3.06 Oe to 41.84, and the magnetic field measurement error also showed a good linear relationship with the diameter *d*${}_{1}$ error y = −1679.7347x − 1.88888, as shown in Figure 10b, $R^{2}$ = 0.99703. In summary, it can be concluded that the preparation error of hole 1 has a great influence on the measurement of the MFI, because the magnetic fluid filled in the hole has a great influence on the coupling of the two fiber cores. Therefore, in the process of device preparation, we should reduce the error of hole 1 as much as possible to improve the measurement accuracy of MFI. In addition, according to the above analysis results, when the error of hole 1 is within the range of ±3% the shift of dip wavelength and the MFI show a good linear relationship, and the sensitivity changes within an acceptable range. Thus, the errors at other MFIs can also be obtained using similar relations.

**Figure 11:**
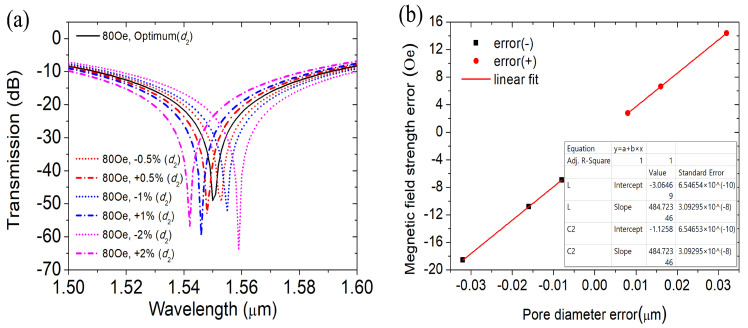
(**a**) Transmission spectra at different errors of *d*${}_{2}$ and (**b**) the relationship between the error of *d*${}_{2}$ and magnetic field error.

As shown in Figure 11a, we also analyze the MFI measurement errors within the range of air hole 2 error ±2% *d*${}_{2}$ with the other optimal structural parameters unchanged. When the fabrication error of pore 2 increases from −0.5% *d*${}_{2}$ to −2% *d*${}_{2}$, the coupling wavelength moves in the direction of long wavelength successively, and the coupling wavelength moves from 1.553 µm to 1.559 µm under the MFI 80 Oe. The results show that there is a good linear relationship between the magnetic field measurement error and the porosity diameter error y = 484.7324x − 3.06469, $R^{2}$ = 1, as shown in Figure 11b. Similarly, when the fabrication error increases from +0.5% *d*${}_{2}$ to +2% *d*${}_{2}$, the coupling wavelength shifts blue from 1.548 µm to 1.542 µm. The measurement error of the corresponding MFI increased from 6.9 Oe to 18.57 Oe, and the magnetic field measurement error also showed a good linear relationship with the error of d${}_{2}$, y = 484.7324x −1258, $R^{2}$ = 1, as shown in the figure. It can be obtained from the above analysis results that the preparation error of hole 2 has less influence on the magnetic field measurement than that of hole 1. Therefore, in the process of device preparation, we should control the error as much as possible within ±0.5% *d*${}_{2}$ to improve the accuracy of MFI measurement. In addition, according to the above analysis, the errors under other magnetic field strengths can also be obtained using similar relations.

**Figure 12:**
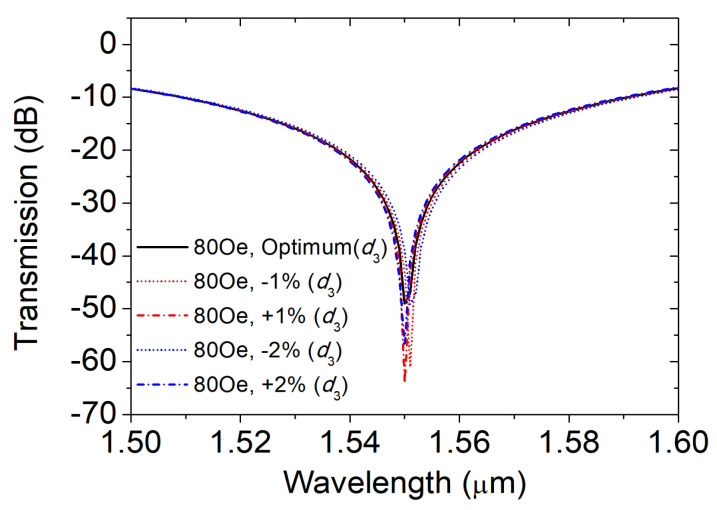
Transmission spectra at different errors of *d*${}_{3}$.

Finally, we analyze the effects of the fabrication errors of the remaining third type of pores on the magnetic field sensing characteristics. As can be seen from Figure 12, when the error of *d*${}_{3}$ changes within the range of ±2% d${}_{3}$, the coupling wavelength hardly changes when the magnetic field is 80 Oe. When the diameter *d*${}_{3}$ error increases from +1% *d*${}_{3}$ to +2% *d*${}_{3}$, the coupling wavelength remains unchanged at 1.55 µm, and there is no MFI measurement error. When the error changes from −1% *d*${}_{3}$ to −2% *d*${}_{3}$, it remains unchanged at 1.551, and the magnetic field strength error is only 3 Oe. Therefore, the third type of air hole has the strongest tolerance for error generated during the production process.

The designed MF-filled DC-PCF is an optical fiber structure consisting of two single-mode fibers (SMFs) spliced together. In the experiment, a broadband light source (BBS) provides continuous light, and the incident light first enters the left SMF, which is an optical fiber capable of transmitting a single mode of light. Then, the incident light passes through the DC-PCF filled with magnetic material and enters the SMF on the right side. In the DC-PCF filled with the MF, the strength of the magnetic field is changed by adjusting the solenoid. The adjusted magnetic field is used to change the refractive index of the MF, thus controlling the propagation of the light and coupling. Finally, the light arrives at the optical spectrum analyzer (OSA), the magnetic field intensity was adjusted, and the transmission spectrum under different magnetic fields in the range of 80–260 Oe was measured and recorded. By analyzing the variation in coupling wavelength with magnetic field, the sensing characteristics of the sensor are obtained.

The comparison of MF sensors using fibers and those using the MF is shown in Table 1. Many sensor types, such as birefringence interference [27], Mach–Zehnder interference [28,29,30], Fabry–Perot interference [31], Sagnac interference [32,33], surface plasmon resonance [34], and directional coupling [35], have been employed for measuring the magnetic field. Several magnetic field sensors [31,35] did not have a linear responses, which reduces the accuracy of the sensor and limits their applications. Due to the strong absorption of light by magnetic fluid, the optical signal will be feeble when light reflects from or passes through the magnetic fluid [28]. Our proposed magnetic field sensor used the directed coupling in MF-filled DC-PCF. A linear response of 80–260 Oe was used to attain the high sensitivity of 524.2 pm/Oe.

## 4. Conclusions

A distinct MF sensor based on the DC-PCF has been developed with a finite element method. The magnetic field may be calculated using the change in the dip wavelength field to be measured. The simulated results show a strong linear link between the magnetic field in the 80–260 Oe region and the inclination wavelength shift. The highest level of sensitivity is 531.81 pm/Oe; while this is going on, the performance of magnetic sensing can be improved any more by modifying the DC-PCF’s structural properties. Our MF-filled DC-PCF can successfully reduces the loss caused by magnetic fluid, showing a good prospect of magnetic field sensing.

## Figures and Tables

**Table 1:** Comparison of magnetic field sensors based on MF-filled optica.

Sensor Type and Reference	Detection Range (Oe)	Linear Fitting	Sensitivity (pm/Oe)
Birefringence Interference [27]	0–450	Yes	24.2
SMF-PCF-SMF [28]	0–450	Yes	24.2
Fabry–Perot interference [31]	0–450	Yes	24.2
SMF-No Core Fiber-SMF [29]	40–100	Yes	90.5
Tapered and Lateral	38–225	Yes	14.1
Spliced SMFs [30]	250–475	Yes	26
Directional Coupling [35]	0–300	Yes	4.8
Sagnac interference [32]	0–2000	No	4.8 (Maximum)
Sagnac interference [33]	410–600	Yes	384
Surface Plasmon Resonance [34]	0–500	Yes	44
Directional Coupling (This work)	80–260	Yes	524.24

## Data Availability

The references contain some of the information that was used. On a fair request, the authors will provide further data.
